# Co-Optimization Operation of Distribution Network-Containing Shared Energy Storage Multi-Microgrids Based on Multi-Body Game

**DOI:** 10.3390/s25020406

**Published:** 2025-01-11

**Authors:** Hao Wu, Ge Cao, Rong Jia, Yan Liang

**Affiliations:** 1 School of Electrical Engineering, Xi’an University of Technology, Xi’an 710048, China; wuhaoacm@xaut.edu.cn; 2 Xi’an Power Supply Company, State Grid Shaanxi Electric Power Co., Ltd., Xi’an 710032, China

**Keywords:** multi-microgrids, distribution network, shared energy storage, one-leader-two-followers, Shapley value method

## Abstract

Under the carbon peaking and carbon neutrality target background, efficient collaborative scheduling between distribution networks and multi-microgrids is of great significance for enhancing renewable energy accommodation and ensuring stable system operation. Therefore, this paper proposes a collaborative optimization method for the operation of distribution networks and multi-microgrids with shared energy storage based on a multi-body game. The method is modeled and solved in two stages. In the first stage, a multi-objective optimization configuration model for shared energy storage among multi-microgrids is established, with optimization objectives balancing the randomness of renewable energy fluctuations and the economics of each microgrid undertaking shared energy storage. The charging and discharging interactive power of energy storage and each microgrid at various time periods are obtained and passed to the second stage. In the second stage, with the distribution network as the leader and shared energy storage and multi-microgrids as followers, a game optimization model with one leader and 2 followers is established. The model is solved based on an outer-layer genetic algorithm nested with an inner-layer solver to determine the electricity purchase and sale prices among the distribution network, multi-microgrids, and shared energy storage at various time periods, thereby minimizing operational costs. Finally, based on the power interaction of microgrids to measure their contributions, an improved Shapley value cost allocation method is proposed, effectively achieving a balanced distribution of benefits among the distribution network, shared energy storage, and multi-microgrids, thereby improving overall operational revenue. Meanwhile, a new method for calculating the shared energy storage capacity and the upper limit of charging and discharging power based on a game framework was proposed, which can save 37.23% of the power upper limit and 44.89% of the capacity upper limit, effectively saving the power upper limit and capacity upper limit.

## 1. Introduction

In the context of the “dual carbon” strategy, integrated energy microgrids have achieved rapid development due to their significant advantages in replacing fossil fuels with clean energy and promoting low-carbon and sustainable energy development. Consequently, the issue of optimal operation of integrated energy microgrids has become a focal point of research [1,2]. As the number of microgrids continues to increase, a new development paradigm of multi-microgrid joint scheduling is gradually emerging [3]. However, the coupling and interaction mechanisms between multi-microgrids and multi-energy flows are complex, and various uncertainties, such as those from energy sources and loads, are superimposed, posing unprecedented challenges to traditional optimal operation methods [4].

Scholars have achieved a series of accomplishments in the field of optimal operation research for multi-microgrids. Ref. [5] established a day-ahead low-carbon economic scheduling model for integrated energy multi-microgrids, aiming to minimize the operational costs of microgrids and promote the low-carbon operation of the system. Ref. [6] proposed a multi-microgrid scheduling strategy for electricity-hydrogen-integrated energy multi-microgrids, considering real-time energy supply and demand states and power interaction states of energy storage. With the advancement of the “dual carbon” goals, the pressure on renewable energy consumption has further increased. As a flexible resource, energy storage can effectively mitigate the intermittency of renewable energy. However, multi-microgrids face high costs in configuring energy storage. In recent years, the concept of shared energy storage has gradually emerged, providing a new approach for the development of multi-microgrid energy storage [7,8]. Ref. [9] proposed an optimal scheduling method for integrated energy multi-microgrids considering shared energy storage, which reduced the operational costs of multi-microgrids by coordinating the interactive electric power between each microgrid and shared energy storage. However, this study set the parameters on the shared energy storage side to fixed values, resulting in overly idealized outcomes. The Ref. [10] established a bi-level optimal configuration model for integrated energy multi-microgrids considering shared energy storage, verifying the economic advantages of multi-microgrid shared energy storage over individually built energy storage by each microgrid. Both Refs. [11,12] introduced demand response mechanisms on the user side, incorporated shared energy storage, and rationally allocated capacity to each microgrid, demonstrating the synergistic effect of shared energy storage and demand response in enhancing the low-carbon economic performance of multi-microgrids. The above literature focuses on the capacity allocation and operational strategies of shared energy storage in multi-microgrid scheduling, failing to consider the issue of benefit allocation between shared energy storage and multi-microgrids, as well as seeking a balance between using energy storage to smooth fluctuations and improving the economy.

When addressing energy trading issues, game theory serves as an effective tool, providing valuable insights into the challenging problem of benefit allocation among multiple entities [13,14]. Ref. [15] proposes an optimal scheduling model for multi-microgrid-shared energy storage systems, utilizing energy storage to smooth out the fluctuations from renewable energy sources integrated into each microgrid and allocating the capacity of shared energy storage in a reasonable way to ensure the maximization of benefits for both individual microgrids and the shared energy storage. The Ref. [16] constructs a cooperative game model encompassing the economic benefits of microgrids and energy storage, aiming to maximize the benefits for all parties involved and achieve a win-win situation. Both of the above-mentioned literature establish traditional game structures for multi-microgrids and shared energy storage, treating each entity as an equally powerful player. However, in real-world scenarios, complex cooperation or competition relationships exist among multiple entities. The Ref. [17] introduces a mixed-game bi-level scheduling model considering the coordination between microgrids and shared energy storage. The upper level establishes a leader-follower game framework with microgrids as leaders and prosumers as followers, while the lower level establishes a cooperative game framework between prosumers and shared energy storage. This model reflects the complex game relationships among microgrids, prosumers, and shared energy storage while ensuring the benefits of multiple entities. However, when exploring the game relationships between multi-microgrids and shared energy storage, the aforementioned literature fails to fully consider the participation of distribution networks and does not fully leverage the characteristics of multi-microgrid grid connection by incorporating the tripartite consideration of shared energy storage, multi-microgrids, and distribution networks into game-based scheduling.

The Shapley value method is a traditional cooperative game allocation method, some studies have proposed a gain allocation model based on the improved Shapley value method by combining the influencing factors of optimal scheduling with the introduction of comprehensive correction coefficients based on the Shapley value method [18], and such improvements can more accurately measure the value of the individual’s contribution, but they cannot overcome the problem of combinatorial explosion inherent in the application of the Shapley value method to large-scale systems, but cannot overcome the combinatorial explosion problem inherent in the Shapley value method when applied to large-scale systems [19]. Cooperative game parsimony algorithms have been proposed for solving the problem of allocating cooperative gains to large-scale interests. The Ref. [20] proposes to utilize the Aumann–Shapley (A–S) value method to allocate the cooperative power gains of a total of 100 hydropower plants in the Brazilian hydropower system; The Ref. [21] applies the maximum–minimum cost remaining saving (MCRS) method to the Yalong River Basin wind-photovoltaic-hydroelectricity-nine interest-body system. The maximum–minimum cost remaining saving method was applied to the problem of gaining electricity allocation of the wind-solar-water-nine benefit system in the Yalong River basin. Compared with the Shapley value method, MCRS is faster and occupies less memory. The above cooperative game parsimony algorithm can better overcome the combinatorial explosion problem in the gain allocation of large-scale subjects of interest and possesses computational efficiency, but it cannot reflect the individual advantages when utilizing the currently proposed efficiency algorithm for gain allocation. The major features of these methods are summarized in Table 1

Based on the problems of the above research, this paper constructs a cooperative optimization and operation method of distribution network-containing shared energy storage multi-microgrids based on a multi-body game, considering the three-way interaction of distribution networks, shared energy storage, and multi-microgrids, and the main contributions are as follows:

(1) A collaborative optimization method for the operation of distribution networks and multi-microgrids with shared energy storage is proposed, based on a multi-body game framework. This method is modeled and solved in two stages. In the first stage, a multi-objective optimization configuration model and its corresponding solution algorithm are established. These are used to determine the charging and discharging powers of energy storage to each microgrid at various time periods. The results from this stage are then passed to the second stage. The use of energy storage helps effectively reduce the fluctuation of net load in microgrids while minimizing the cost of shared energy storage borne by each microgrid. This achieves a balance between smoothing renewable energy fluctuations and enhancing economic benefits among multi-microgrids.
(2) In the second stage of the proposed method, a game structure is formed with one leader (the distribution network) and 2 followers (shared energy storage and multi-microgrids). This structure ensures that the scheduling of the three parties can achieve collaboration and equitable distribution of benefits. Consequently, the overall performance is enhanced, promoting effective and equitable operation within the distribution network and multi-microgrids system.
(3) A new method for calculating the capacity of shared energy storage and the upper limits of charging and discharging powers based on a game-theoretic framework is proposed. Compared to the traditional approach of summing up the individually set storage capacities for each microgrid, the new method can more effectively utilize the upper limits of storage power and capacity, thereby enhancing resource utilization efficiency.
(4) An improvement to the Shapley value method is proposed, where the interactive power among microgrids is used as a basis to measure their contributions to the alliance. This improvement can more accurately reflect the individual advantages of microgrids within the alliance and provides a new perspective for addressing the issue of revenue allocation among multi-microgrids.

This paper is constituted of 6 sectors. The multi-objective optimization-based configuration of shared energy storage for multi-microgrids will be stated in Section 2. Section 3 describes a scheduling model of distribution networks with multi-microgrids containing shared energy storage based on a one-leader-two-follower game. Section 4 presents a calculation method of the capacity of shared energy storage and power upper limits for charging and discharging of shared energy storage in a gaming framework. Section 5 gives the case analysis. This paper will provide a comprehensive conclusion in Section 6.

## 2. Multi-Objective Optimization-Based Configuration of Shared Energy Storage for Multi-Microgrids

In the operation of microgrids, there are complex cost and economic trade-offs involved between the deployment of energy storage devices and the regulation of power fluctuations. If the focus is on smoothing renewable energy power fluctuations, sufficient installed capacity of energy storage devices needs to be configured for the microgrid, which increases investment costs. Conversely, if the primary concern is economic efficiency, with only limited smoothing of power fluctuations, the stability and reliability of microgrid operation may be compromised. To effectively mitigate this contradiction, this section proposes a multi-objective optimization configuration model for multi-microgrid shared energy storage that takes into account both the investment cost of energy storage and load fluctuations. The aim is to strike a balance between enhancing economic efficiency and smoothing power fluctuations.

**Figure 1:**
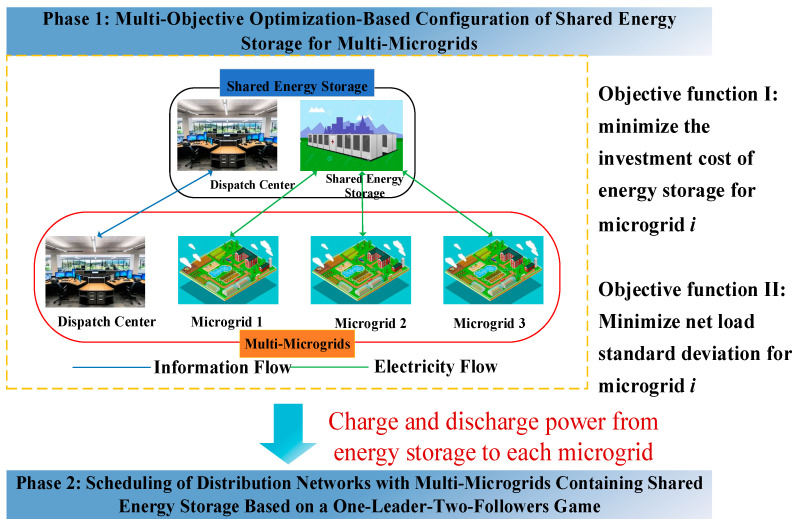
Schematic diagram of multi-microgrids energy storage configuration with shared energy storage.

In the first phase, the multi-microgrids and shared energy storage are considered a single interest entity, where the microgrids bear the investment costs of the energy storage and utilize the shared energy storage to smooth out net load fluctuations. Under the premise of satisfying the coupling constraints between each microgrid and the shared energy storage, the charging and discharging powers of the shared energy storage for each microgrid are optimized. Figure 1 illustrates the schematic diagram of the energy storage configuration for multi-microgrids with shared energy storage.

### 2.1. Objective Function

I. Minimizing energy storage investment costs of microgrid *i*

(1) $$C_{\mathrm{SES},i}=\min(C_{\mathrm{INV},i}+C_{\mathrm{OM},i})$$

where *C*_SES,*i*_ is the energy storage investment cost of microgrid *i*, RMB. *C*_INV,*i*_ is the energy storage investment cost borne by microgrid *i*, and *C*_OM,*i*_ is the energy storage O&M cost borne by microgrid *i*.

The formula for calculating each cost is shown below:

(1) The cost of energy storage investments borne by microgrids *i*, irrespective of the effects of economic development and inflation:

(2) $$C_{\mathrm{INV},i}=\frac{\lambda_{P}P_{\mathrm{SES},i}^{\max}+\lambda_{E}E_{\mathrm{SES},i}^{\max}}{T_{E}}$$

where λ_P_ and λ_E_ denote the unit power investment cost and unit capacity investment cost of shared energy storage, respectively; $P_{\mathrm{SES},i}^{\max}$ and $E_{\mathrm{SES},i}^{\max}$ denote the maximum value of microgrid *i*’s demand for storage power and capacity, respectively; and *T*_E_ denotes the service life of shared energy storage, h.

(2) The cost of energy storage O&M borne by microgrid *i*

(3) $$C_{\mathrm{OM},i}=\sum_{t=1}^{T}[\delta_{\mathrm{OM}}\cdot (P_{\mathrm{SES},C,i}(t)+P_{\mathrm{SES},D,i}(t))\Delta t]$$

where *P*_SES,C,*I*_ (*t*) and *P*_SES,D,*I*_ (*t*) denote the charging and discharging power of the energy storage to microgrid *i* at time *t*, respectively, kW; *δ*_OM_ is the operation and maintenance cost per unit of power for microgrid *i*, RMB. Both *P*_SES,C_(*t*) and *P*_SES,D_(*t*) are measured by sensors on the transmission line between the MMG and the SES.

II. Minimize the standard deviation of the net load of microgrid *i*

(4) $$\begin{array}{c} S_{i}=\sqrt{\frac{1}{T}\sum_{\iota =1}^{T}{\left[P_{\mathrm{load},i}(t)-P_{\mathrm{WT},i}(t)-P_{\mathrm{PV},i}(t)+P_{\mathrm{SES},C,i}(t)-P_{\mathrm{SES},D,i}(t)-{\bar{P}}_{\mathrm{load},i}\right]}^{2}} \end{array}$$

(5) $${\bar{P}}_{\mathrm{load},i}=\frac{1}{T}\sum_{t=1}^{T}[P_{\mathrm{load},i}(t)-P_{\mathrm{WT},i}(t)-P_{\mathrm{PV},i}(t)+P_{\mathrm{SES},C,i}(t)-P_{\mathrm{SES},D,i}(t)]\Delta t$$

where *S_i_* is the standard deviation of net load of microgrid *i*; *P*_load,*i*_(*t*) is the load value of microgrid *i* at time *t*; *P*_WT,*i*_(*t*) and *P*_PV,*i*_(*t*) are the wind and PV power consumed by microgrid *i* at time *t*, respectively; and ${\bar{P}}_{\mathrm{load},i}$ is the average value of the equivalent load of microgrid *i*.

### 2.2. Constraints

(1) Coupling constraints on the sharing of energy storage and microgrid *i*

(6) $$\left\{\begin{array}{l} 0\leq P_{\mathrm{SES},C,i}(t)\leq U_{\mathrm{SES},C,i}(t)P_{\mathrm{SES},C,i}^{\max}\\ 0\leq P_{\mathrm{SES},D,i}(t)\leq U_{\mathrm{SES},D,i}(t)P_{\mathrm{SES},D,i}^{\max}\\ U_{\mathrm{SES},C,i}(t)+U_{\mathrm{SES},D,i}(t)\leq 1 \end{array}\right.$$

(7) $$P_{\mathrm{SES},i}^{\max}=\max[P_{\mathrm{SES},C,i}(t),P_{\mathrm{SES},D,i}(t)],t=1,\cdots ,T$$

where *P*_SES,C,*i*_(*t*), *P*_SES,D,*i*_(*t*) denote the charging and discharging power of the energy storage to microgrid *i* at time *t*, kW; $P_{\mathrm{SES},C,i}^{\max}$, $P_{\mathrm{SES},D,i}^{\max}$ denote the upper limit of the charging and discharging power of the energy storage to microgrid *i*; *U*_SES,C,*i*_(*t*), *U*_SES,D,*i*_(*t*) ensure that the energy storage can only charge or discharge the microgrid *i* at the same time in a scheduling cycle, and *U*_SES,C,*i*_(*t*) is 1 for charging and 0 for discharging; $P_{\mathrm{SES},i}^{\max}$ is the upper limit of the power demand of microgrid *i* for energy storage.

(2) Charge and discharge power constraints

(8) $$E_{i}(t)=E_{i}(t-1)+(\eta_{\mathrm{SES},C}P_{\mathrm{SES},C,i}(t)-P_{\mathrm{SES},D,i}(t)/\eta_{\mathrm{SES},D})\Delta t$$

(9) $$\sum_{t=1}^{T}(\eta_{\mathrm{SES},C}P_{\mathrm{SES},C,i}(t)-P_{\mathrm{SES},D,i}(t)/\eta_{\mathrm{SES},D})=0$$

(10) $$E_{\mathrm{SES},i}^{\max}=\mu_{E}\cdot \max(E_{i}(t)),t=1,\cdots ,T$$

where *η*_SES,C_, *η*_SES,D_ denote the charging and discharging efficiencies of energy storage, respectively; *E_i_*(*t*) denotes the storage capacity required for charging and discharging energy storage to microgrid *i*, kW·h; $E_{\mathrm{SES},i}^{\max}$ is the upper limit of the capacity demand of energy storage utilized by microgrid *i*, kW·h, and *µ*_E_ is the capacity margin of the microgrid shared energy storage.

## 3. Scheduling of Distribution Networks with Multi-Microgrids Containing Shared Energy Storage Based on a One-Leader-Two-Followers Game

### 3.1. A One-Leader-Two-Followers Game Scheduling Framework

In the system, the distribution network, multi-microgrids, and shared energy storage represent different stakeholders, each pursuing optimal objectives and striving to avoid unfavorable strategies. Ultimately, through negotiation, they reach a compromise and form a comprehensive operation scheme acceptable to all parties. Therefore, in the second stage, a one-leader-two-followers game model for the distribution network and multi-microgrids with shared energy storage is established, where the distribution network serves as the leader, setting the purchase and sale prices of electricity from multi-microgrids and shared energy storage based on its own load demand, effectively incentivizing microgrids to participate in demand response. If there is still a power shortage, it purchases electricity from the main grid.

**Figure 2:**
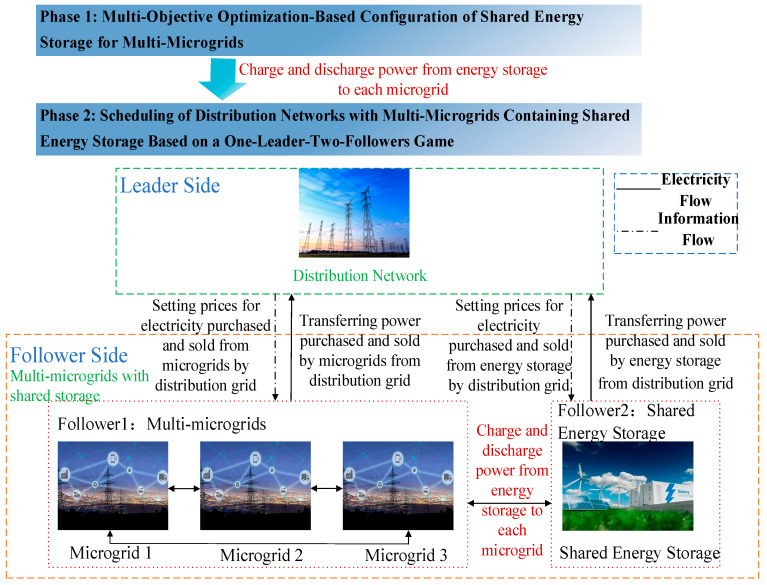
A master-two-slave game scheduling framework.

The multi-microgrids and shared energy storage play the role of followers. Firstly, the microgrids utilize shared energy storage to stabilize net load fluctuations in the first stage. Subsequently, they prioritize using their own power generation units and power exchange with other microgrids to meet load demands. They can also flexibly adjust their purchase and sale power with the distribution network based on the prices set by the distribution network. The shared energy storage first achieves power exchange with each microgrid based on the charging and discharging power calculated in the first stage. If the shared energy storage capacity is insufficient or surplus, it meets the demand by purchasing or selling electricity from the distribution network to achieve power balance. The scheduling framework for the three parties is shown in Figure 2.

### 3.2. Leader Scheduling Model of Distribution Network

1. Objective function

The decision variable for optimal scheduling of the distribution network is the purchase and sale price of electricity, and the objective of optimal operation is to minimize the total cost *C*_DN_:

(11) $$C_{\mathrm{DN}}=\min(C_{\mathrm{grid}}+C_{\mathrm{MMG},\mathrm{DN}}+C_{\mathrm{SES},\mathrm{DN}})$$

where C_DN_ is the total cost of the distribution network, RMB; C_grid_ is the cost of power purchased by the distribution network from the main grid; C_MMG,DN_ is the cost of power interaction between the distribution network and multi-microgrids; and C_SES,DN_ is the cost of power interaction between the distribution network and shared energy storage. The formula for calculating each cost is as follows:

(1) Cost of electricity purchased from the main grid:

(12) $$C_{\mathrm{grid}}=\sum_{t=1}^{T}[\delta_{\mathrm{sell},\mathrm{grid}}(t)P_{\mathrm{buy},\mathrm{DN}}(t)]\Delta t$$

where *δ*_sell,grid_(*t*) is the selling price of the main grid, RMB; *P*_buy,DN_(*t*) is the power purchased from the main grid by the distribution network at time *t*, kW.

(2) Cost of power interaction with multi-microgrids

(13) $$C_{\mathrm{MMG},\mathrm{DN}}=\sum_{i=1}^{N}\sum_{t=1}^{T}\left[\delta_{\mathrm{buy},\mathrm{DN}}(t)P_{\mathrm{sell},i}(t)-\delta_{\mathrm{sell},\mathrm{DN}}(t\right)P_{\mathrm{buy},i}(t)]\Delta t$$

where *δ*_buy,DN_(*t*) is the purchased power price of the optimized distribution network, RMB; *δ*_sell,DN_(*t*) is the sold power price of the optimized distribution network; *P*_buy,*i*_(*t*) and *P*_sell,*i*_(*t*) are the purchased and sold power of microgrid *i* from the distribution network at time *t*. Both *P*_buy,*i*_(*t*) and *P*_sell,*i*_(*t*) are measured by sensors on the transmission line between the MMG and the DN.

(3) Electricity interaction costs with shared energy storage

(14) $$C_{\mathrm{SES},\mathrm{DN}}=\sum_{t=1}^{T}\left[\delta_{\mathrm{buy},\mathrm{DN}}(t)P_{\mathrm{sell},\mathrm{SES}}(t)-\delta_{\mathrm{sell},\mathrm{DN}}(t\right)P_{\mathrm{buy},\mathrm{SES}}(t)]\Delta t$$

where *P*_buy,SES_(*t*), *P*_sell,SES_(*t*) are the power purchased and sold from the distribution network by the shared energy storage at time *t*, kW.

2. Constraints

The constraints include tidal balance constraints, state variable constraints, access to distributed energy sources constraints, and time-of-use tariff constraints, as shown below:

(1) Tidal balance constraints

(15) $$\left\{\begin{array}{l} P_{p}=U_{p}\sum_{q\in p}U_{q}\left(G_{pq}\cos\delta_{pq}+B_{pq}\sin\delta_{pq}\right)(p=1,2,\cdots ,n)\\ Q_{p}=U_{p}\sum_{q\in p}U_{q}\left(G_{pq}\sin\delta_{pq}-B_{pq}\cos\delta_{pq}\right)(p=1,2,\cdots ,n) \end{array}\right.$$

where *n* is the number of nodes in the distribution network; *P_p_*(kW), *Q_p_*(kVar), *U_p_*(kV) are the active power, reactive power and voltage magnitude injected at node *p*, respectively; *G_pq_*(S), *B_pq_*(S), *δ_pq_*(rad) are the conductance, conductance, and voltage phase difference between nodes *p* and *q*, respectively.

(2) State variable constraints

(16) $$\left\{\begin{array}{l} U_{p}^{\min}\leq U_{p}\leq U_{p}^{\max}\\ \left|\delta_{p}-\delta_{q}\right|\leq {\left|\delta_{p}-\delta_{q}\right|}^{\max}\\ 0\leq S_{l}\leq S_{l}^{\max} \end{array}\right.$$

where $U_{p}^{\max}$, $U_{p}^{\min}$ are the upper and lower limits of the voltage amplitude injected at node *p*, respectively, kV; *G_pq_*, *B_pq_*, and *δ_pq_* are the conductance, conductivity, and voltage phase difference between nodes *p* and *q*, respectively; and *S_l_* and $S_{l}^{\max}$ are the power and the upper limit of the power of the distribution branch *l*, respectively, kVA.

(3) Connecting to distributed energy constraints

(17) $$\left\{\begin{array}{l} 0\leq P_{\mathrm{DG}}(t)\leq P_{\mathrm{DG}}^{\max}\\ 0\leq Q_{\mathrm{DG}}(t)\leq Q_{\mathrm{DG}}^{\max} \end{array}\right.$$

where *P*_DG_(*t*), *Q*_DG_(*t*) are the active and reactive power output of distributed energy at time *t*, kW; $P_{\mathrm{DG}}^{\max}$, $P_{\mathrm{DG}}^{\min}$ are the upper and lower limits of the active power output of distributed energy; $Q_{\mathrm{DG}}^{\max}$, $Q_{\mathrm{DG}}^{\min}$ are the upper and lower limits of the reactive power output of distributed energy, respectively, kVar.

(4) Time-of-use tariff constraints

The purchase and sale price of electricity with microgrids and shared energy storage set by the distribution network is a time-of-day tariff, and the sale price and purchase price of electricity in the valley, normal and peak hours, satisfy the following relational equation:

(18) $$\delta_{\mathrm{buy},\mathrm{DN}}(t)=\left\{\begin{array}{l} \delta_{\mathrm{buyv},\mathrm{DN}}(t),t\in T_{v}\\ \delta_{\mathrm{buyf},\mathrm{DN}}(t),t\in T_{f}\\ \delta_{\mathrm{buyp},\mathrm{DN}}(t),t\in T_{p} \end{array}\right.,\delta_{\mathrm{buyv},\mathrm{DN}}(t)<\delta_{\mathrm{buyf},\mathrm{DN}}(t)<\delta_{\mathrm{buyp},\mathrm{DN}}(t)$$

(19) $$\delta_{\mathrm{sell},\mathrm{DN}}(t)=\left\{\begin{array}{l} \delta_{\mathrm{sellv},\mathrm{DN}}(t),t\in T_{v}\\ \delta_{\mathrm{sellf},\mathrm{DN}}(t),t\in T_{f}\\ \delta_{\mathrm{sellp},\mathrm{DN}}(t),t\in T_{p} \end{array}\right.,\delta_{\mathrm{sellv},\mathrm{DN}}(t)<\delta_{\mathrm{sellf},\mathrm{DN}}(t)<\delta_{\mathrm{sellp},\mathrm{DN}}(t)$$

where *T*_v_, *T*_f_, and *T*_p_ are the valley time, the usual time, and the peak time, respectively; h; *δ*_buyv,DN_(*t*), *δ*_buyf,DN_(*t*) and *δ*_buyp,DN_(*t*) are the distribution network purchase price in the valley time, the usual time, and the peak time, respectively, in RMB; *δ*_sellv,DN_(*t*), *δ*_sellf,DN_(*t*) and *δ*_sellp,DN_(*t*) are the distribution network sale price in the valley time, the usual time, and the peak time, respectively.

In order to ensure that the distribution network is profitable, the selling price of electricity in the distribution network must be higher than the purchasing price of electricity in all time periods:

(20) $$\left\{\begin{array}{l} \delta_{\mathrm{sellv},\mathrm{DN}}(t)>\delta_{\mathrm{buyv},\mathrm{DN}}(t),t\in T_{v}\\ \delta_{\mathrm{sellf},\mathrm{DN}}(t)>\delta_{\mathrm{buyf},\mathrm{DN}}(t),t\in T_{f}\\ \delta_{\mathrm{sellp},\mathrm{DN}}(t)>\delta_{\mathrm{buyv},\mathrm{DN}}(t),t\in T_{p} \end{array}\right.$$

In order to prevent the subject distribution network from raising the electricity sales price for its own benefit during the gaming process, the average electricity sales price during the scheduling period *T* should be no greater than the upper limit of the electricity sales price of the distribution network:

(21) $$\frac{1}{T}\sum_{t=1}^{T}\delta_{\mathrm{sell},\mathrm{DN}}(t)\leq \delta_{\mathrm{sell},\mathrm{DN}}^{\max}$$

where $\delta_{\mathrm{sell},\mathrm{DN}}^{\max}$ is the upper limit of the selling price of electricity from the distribution network.

### 3.3. Follower Scheduling Model of Multi-Microgrids with Shared Energy Storage

#### 3.3.1. Shared Energy Storage Scheduling Model

1. Objective function

The charging and discharging power *P*_SES,C,*i*_(*t*), *P*_SES,D,*i*_(*t*) of the shared storage to microgrid *i* are optimized in the first stage, so the decision variable of the shared storage scheduling model is the interacting power with the distribution network, and the objective of the optimized operation is to minimize the total cost *C*_SES_:

(22) $$C_{\mathrm{SES}}=\min(C_{\mathrm{INV}}+C_{\mathrm{OM}}-C_{\mathrm{SES},\mathrm{DN}})$$

where C_SES_ is the total cost of shared energy storage, RMB; C_INV_ is the investment cost of shared energy storage; C_OM_ is the operation and maintenance cost of shared energy storage; and C_SES,DN_ is the cost of electricity interaction between shared energy storage and the distribution network. The formula for each cost is as follows:

(1) Cost of investment in shared energy storage

(23) $$C_{\mathrm{INV}}=\frac{\lambda_{P}P_{\mathrm{SES}}^{\max}+\lambda_{E}E_{\mathrm{SES}}^{\max}}{T_{E}}$$

where λ_P_ and λ_E_ are the unit power investment cost and unit capacity investment cost of shared energy storage, respectively, in RMB; $P_{\mathrm{SES},i}^{\max}$ and $E_{\mathrm{SES},i}^{\max}$ are the power ceiling and capacity ceiling of shared energy storage, respectively; *T*_E_ is the service life of shared energy storage, in h.

(2) Cost of operation and maintenance of shared energy storage

(24) $$C_{\mathrm{OM}}=\sum_{t=1}^{T}[\delta_{\mathrm{OM}}\cdot (P_{\mathrm{SES},C}(t)+P_{\mathrm{SES},D}(t))\Delta t]$$

where *P*_SES,C_(*t*) and *P*_SES,D_(*t*) denote the charging and discharging power of the energy storage at time *t*, respectively, RMB; *δ*_OM_ is the unit power operation and maintenance cost of the energy storage.

(3) Cost of power interaction with the distribution network (Equation (14))

2. Constraints

(1) Shared storage constraints, which include the inherent constraints on charging and discharging of shared storage, the upper and lower power limit constraints, and the power relationship constraints between two neighboring time periods, are as follows:

(25) $$\left\{\begin{array}{l} E_{\mathrm{SES}}(0)=E_{\mathrm{SES}}(24)=20\%E_{\mathrm{SES}}^{\max}\\ 10\%E_{\mathrm{SES}}^{\max}\leq E_{\mathrm{SES}}(t)\leq 90\%E_{\mathrm{SES}}^{\max}\\ E_{\mathrm{SES}}(t)=E_{\mathrm{SES}}(t-1)+(\eta_{\mathrm{SES},C}P_{\mathrm{SES},C}(t)-\frac{1}{\eta_{\mathrm{SES},D}}P_{\mathrm{SES},D}(t))\Delta t\\ 0\leq P_{\mathrm{SES},C}(t)\leq U_{\mathrm{SES},C}(t)P_{\mathrm{SES}}^{\max}\\ 0\leq P_{\mathrm{SES},D}(t)\leq U_{\mathrm{SES},D}(t)P_{\mathrm{SES}}^{\max}\\ U_{\mathrm{SES},C}(t)+U_{\mathrm{SES},D}(t)\leq 1 \end{array}\right.$$

where *E*_SES_(*t*) denotes the capacity of shared energy storage at time *t*; *η*_SES,C_, *η*_dSES,D_ denote the charging and discharging efficiency of the energy storage, respectively; *P*_SES,C_(*t*), *P*_SES,D_(*t*) denote the charging and discharging power of the energy storage, respectively; *E*_SES_(0) and *E*_SES_(24) denote the energy stored in the beginning and the end of the scheduling cycle, respectively; $E_{\mathrm{SES}}^{\max}$ denotes the upper limit of the storage capacity of the energy storage; $P_{\mathrm{SES}}^{\max}$ denotes the upper limit of the charging and discharging power of the energy storage; *U*_SES,C_(*t*) and *U*_SES,D_(*t*) ensure that the energy storage can only be charged or discharged at the same moment in the scheduling cycle, and *U*_SES,C_(*t*) of 1 denotes charging, and *U*_SES,C_(*t*) of 0 denotes discharging.

(2) Shared storage power balance constraints, where the charging and discharging power *P*_SES,C,*i*_ (*t*), *P*_SES,D,*i*_(*t*) of the shared storage to microgrid i are derived from the optimized scheduling results in the first stage.

(26) $$\sum_{i=1}^{N}\left[P_{\mathrm{SES},C,i}(t)-P_{\mathrm{SES},D,i}(t)\right]+U_{\mathrm{buy},\mathrm{SES}}(t)P_{\mathrm{buy},\mathrm{SES}}(t)-U_{\mathrm{sell},\mathrm{SES}}(t)P_{\mathrm{sell},\mathrm{SES}}(t)=P_{\mathrm{SES},C}(t)-P_{\mathrm{SES},D}(t)$$

where *U*_buy,SES_(*t*), *U*_sell,SES_(*t*) ensures that the energy storage can only purchase or sell electricity with the distribution network at the same moment in the scheduling cycle, and *U*_buy,SES_(*t*) of 1 indicates the purchase of electricity, and *U*_buy,SES_(*t*) of 0 indicates the sale of electricity. *P*_buy,SES_(*t*) and *P*_sell,SES_(*t*) are the power purchased and sold by the shared storage from the distribution network at time *t*, respectively.

#### 3.3.2. Multi-Microgrids Scheduling Model

1. Objective function

(27) $$C_{\mathrm{MMG}}=\min(C_{\mathrm{SCU}}-C_{\mathrm{MMG},\mathrm{DN}})$$

where C_MMG_ is the total cost of the multi-microgrid; C_SCU_ is the cost of starting and stopping coal consumption of small coal-fired units; C_MMG,DN_ is the cost of power interaction between the multi-microgrid and the distribution network. The formula for each cost is as follows:

(1) Startup and shutdown and coal costs for small coal-fired units

(28) $$\begin{array}{l} C_{\mathrm{SCU}}=\sum_{i=1}^{N}[\sum_{t=1}^{T}\left(a_{1}P_{\mathrm{SCU},i}^{2}(t)+b_{1}P_{\mathrm{SCU},i}(t)+c_{1}P_{\mathrm{SCU},i}^{2}(t)\right)\\ +\delta_{\mathrm{SCU}}\sum_{t=1}^{T}\left[U_{\mathrm{SCU},i}(t+1)(1-U_{\mathrm{SCU},i}(t))+U_{\mathrm{SCU},i}(t)(1-U_{\mathrm{SCU},i}(t+1))]\right] \end{array}$$

where *P*_SCU,*i*_(*t*) is the scheduling output of small and medium-sized coal-fired units of microgrid *i* at time *t*; *a*_1_, *b*_1_, and *c*_1_ are the coal consumption coefficients of the small-sized coal-fired unit, respectively; *U*_SCU,*i*_(*t*) is a binary variable, *U*_SCU,*i*_(*t*) = 1 indicates startup, and *U*_SCU,*i*_(*t*) indicates shutdown; and *δ*_SCU_ is the startup and shutdown cost coefficient of the unit.

(2) Cost of power interaction with the distribution network (Equation (13))

2. Constraints

(1) Operational constraints for small coal-fired units

(29) $$\left\{\begin{array}{l} P_{\mathrm{SCU}}^{\min}\leq P_{\mathrm{SCU},i}(t)\leq P_{\mathrm{SCU}}^{\max}\\ P_{\mathrm{SCU}}^{\mathrm{down}}\leq P_{\mathrm{SCU},i}(t)-P_{\mathrm{SCU},i}(t-1)\leq P_{\mathrm{SCU}}^{\mathrm{up}} \end{array}\right.$$

where $P_{\mathrm{TU}}^{\max}$, $P_{\mathrm{TU}}^{\min}$ are the upper and lower limits of output power of small coal-fired units; $P_{\mathrm{TU}}^{\mathrm{up}}$, $P_{\mathrm{TU}}^{\mathrm{down}}$ are the upper and lower limits of output power of small coal-fired units.

(2) Electricity trading constraints between microgrids

(30) $$-P_{e,i}^{\max}\leq P_{ij}(t)\leq P_{e,i}^{\max}$$

where $P_{e,i}^{\max}$ is the upper limit of the electric power of microgrid *i* interacting with other microgrids.

(3) Power balance constraints for microgrids

(31) $$P_{\mathrm{SCU},i}(t)+P_{\mathrm{WT},i}(t)+P_{\mathrm{PV},i}(t)+U_{\mathrm{buy},i}(t)P_{\mathrm{buy},i}(t)-U_{\mathrm{sell},i}(t)P_{\mathrm{sell},i}(t)+P_{\mathrm{SES},D,i}(t)-P_{\mathrm{SES},C,i}(t)=P_{\mathrm{load},i}(t)+P_{ij}(t)$$

where *P_ij_*(*t*) is the interaction power between microgrids *i* and *j* at time period *t*, *P_ij_*(*t*)> 0 indicates that microgrid *i* sells power to microgrid *j*, and *U*_buy,*i*_(*t*) and *U*_sell,*i*_(*t*) ensure that the microgrids can interact with the distribution network only at the same moment in the scheduling cycle. *U*_buy,*i*_(*t*) is 1 for power purchase and 0 for power sale. *P_ij_*(*t*) is measured by sensors on the transmission lines between microgrids. *P*_SCU,*i*_(*t*), *P*_WT,*i*_(*t*) and *P*_PV,*i*_(*t*) are measured by sensors on small coal-fired units, WTs, and PVs, respectively.

(4) Power constraints on the liaison line

(32) $$-P_{\mathrm{LL},i}^{\max}\leq (U_{\mathrm{buy},i}(t)P_{\mathrm{buy},i}(t)-U_{\mathrm{sell},i}(t)P_{\mathrm{sell},i}(t)+P_{\mathrm{SES},D,i}(t)-P_{\mathrm{SES},C,i}(t)-P_{ij}(t))\leq P_{\mathrm{LL},i}^{\max}$$

where $P_{\mathrm{LL},i}^{\max}$ is the maximum transmission power of the contact line.

### 3.4. Cost-Sharing Method for Each Microgrid Based on the Improved Shapley Value Method

The Shapley value method is a commonly used cost-sharing method. If the total cost _CMMG_ of multi-microgrids is shared based on the conventional Shapley value method, the cost shared by microgrid *i* can be expressed as follows:

(33) $$C_{i}=\sum_{s\in S_{i}}\frac{(|s|-1)!(N-|s|)!}{N!}[\nu (s)-\nu (s\backslash \{i\})]$$

where *N* is the number of microgrids; *S_i_* denotes the set consisting of all subsets containing microgrid *i* (*S_i_ =* {1,2,…,*N*}), *|s|* denotes the number of microgrids contained in coalition *s*; *v*(*s*) denotes the cost incurred by coalition *s*; and *v*(*s*\{*i*}) denotes the cost incurred by coalition *s* after coalition *s* has removed microgrid *i*.

As can be seen from Equation (33), the cost-sharing weight of each microgrid in the conventional Shapley value method is fixed, which essentially sets the weight shared by the *N* microgrids to *1/N*. This conventional sharing method is more ideal, ignores the uniqueness of each participant, and fails to adequately take into account the actual contribution of each microgrid to the coalition. Considering that the literature [4] has demonstrated that increasing the power interaction between multi-microgrids can improve the energy efficiency of multi-microgrids, in order to encourage the power interaction between multi-microgrids, this paper proposes an improved Shapley value method, i.e., to measure the contribution to the coalition by using the interaction power *P_ij_*(*t*) between microgrid *i* and microgrid *j*, which then redefines the apportionment weight of microgrid *B_i_*:

(34) $$B_{i}=\frac{\sum_{t=1}^{T}\sum_{\begin{array}{l} j=1\\ j\neq i \end{array}}^{S}P_{ij}(t)}{\sum_{t=1}^{T}\sum_{i=1}^{S}\sum_{\begin{array}{l} j=1\\ j\neq i \end{array}}^{S}P_{ij}(t)}$$

The sum of the weights of the *N* microgrids satisfies $\sum_{i=1}^{N}B_{i}=1$. The difference between the conventional and improved weights of microgrid *i* is $\Delta B_{i}=B_{i}-1/N$. Using the fact that the apportionment result of the conventional Shapley value method can be corrected by an amount of $\Delta B_{i}$, the apportioned cost of microgrid *i* after correction is as follows:

(35) $$C_{i}^{*}=C_{i}+\Delta B_{i}v(s)$$

The proposed weight calculation method can accurately reflect the contribution of each microgrid, thus effectively avoiding the problem of uneven or unreasonable weight distribution and ensuring the objectivity and accuracy of the assessment results.

### 3.5. Solution Methods

The first stage is solved using a multi-objective particle swarm algorithm to optimize the charging and discharging power from the energy storage to each microgrid, and then this optimization result is passed to the second stage. The second stage is solved using a genetic algorithm nested solver. The specific realization process is as follows:

Stage 1: The multi-objective particle swarm algorithm has the advantages of a simple coding method, being easy to implement, and adaptive search, so the algorithm is used to solve the multi-microgrid shared energy storage configuration model. The specific solution process is as follows:

Introduction to the Principle of the Multi-Objective Particle Swarm Algorithm: In the multi-objective particle swarm algorithm, each particle (i.e., a potential solution) is iteratively updated based on the historical experience of itself and its neighboring particles and constantly explores the optimal solution under multiple objective functions in the search space. Through this approach, we can effectively determine the optimal solution for energy storage configuration, which in turn improves the operational efficiency and stability of multi-microgrids. Similar to the single-objective particle swarm algorithm, the multi-objective particle swarm algorithm particle update process is as follows:

(36) $$\begin{array}{l} S_{i+1}=w\times S_{i}+b_{1}\times \mathrm{rand}\times ({\mathrm{ind}}_{i}-L_{i})+b_{2}\times \mathrm{rand}\times (\sec_{i}-L_{i})\\ L_{i+1}=L_{i}+S_{i+1} \end{array}$$

where *S_i_* is the velocity of the particle; *L_i_* is the position of the particle; ind*_i_* is the individual extreme point of each particle; sec*_i_* is the global extreme point of all the particles in the whole population; rand is a random number between 0 and 1; *b*_1_ and *b*_2_ are the learning factors, which are usually taken as *b*_1_ = *b*_2_ = 2.

Given the lack of specific bias in each objective function, this paper adopts a balanced decision-making mechanism to normalize the values of the multi-objective functions of the Pareto optimal solutions and assign the same weighting, integrating them into a single indicator for comparison. Each objective fitness function for each Pareto optimal solution is calculated based on the following formula:

(37) $$\begin{array}{l} f_{l,j}=\frac{g_{l,j}-g_{\min,j}}{g_{\max,j}-g_{\min,j}}\\ g_{l,1}=C_{\mathrm{SES},i},g_{l,2}=S_{i} \end{array}$$

where *f_l_*_,*j*_ is the *j*th fitness value of the *l*th Pareto optimal solution; *g_l_*_,*j*_ is the *j*th objective function value of the *l*th Pareto optimal solution; *g*_max,*j*_ and *g*_min,*j*_ are the maximum and minimum values of the *j*th objective function in the set of Pareto optimal solutions, respectively. For the *l*th Pareto optimal solution, its normalized fitness function *f_l_* is as follows:

(38) $$f_{l}=\sum_{j=1}^{Q}f_{l,j}$$

where *Q* is the dimension of the objective function. The Pareto optimal solution with the smallest standardized fitness function *f_l_* is selected as the optimal compromise solution so as to effectively determine the optimal allocation scheme of energy storage and then improve the operation efficiency and stability of multi-microgrids.

The multi-objective particle swarm algorithm utilizes the dominant relationship among solutions to manage the external archive, ensuring that the solution set in the archive remains efficient and high-quality. The definition of the dominance relationship is as follows: When comparing two solutions, A and B, if A performs no worse than B on all objective functions and is superior to B on at least one objective, then solution A is said to dominate solution B. Conversely, if B dominates A, then B is the dominant solution. If no dominance relationship exists between the two solutions, they are considered to be in a non-dominance relationship. At the initial stage of the algorithm, all particles are initially placed into the external archive. As the algorithm progresses, whenever a new solution is generated, it is compared with the existing solutions in the archive. If the new solution dominates a solution in the archive, the dominant solution in the archive is removed, and the new solution takes its place. If no solution in the archive can dominate the new solution, the new solution is added to the archive. Whenever the velocity and position of the particles are updated, the new solutions continue to be compared and updated with the solutions in the archive according to the aforementioned rules, thereby maintaining an efficient set of non-dominated solutions.

Stage 2: The charging and discharging power *P*_SES,C,*i*_(*t*), *P*_SES,D,*i*_(*t*) of the shared energy storage to microgrid *i* obtained from the solution of Stage 1 are substituted into the one-leader-two-followers game scheduling model in Section 3, which can be expressed as follows:

(39) $$G=\{O;\delta_{\mathrm{DN}}(t);\{P_{i}(t),P_{\mathrm{SES}}(t)\};C_{\mathrm{DN}};\{C_{\mathrm{SES}},C_{\mathrm{MMG}}\}\}$$

The relevant variables in the model are explained as follows:

(1) Distribution networks, shared energy storage, and multi-microgrids form the set of game participants *O*;
(2) Decision variables: the distribution network decision variable is the time-of-day tariff [*δ*_DN_(*t*) = (*δ*_buy,DN_(*t*),*δ*_sell,DN_(*t*))]; the multi-microgrids decision variable is the purchased and sold power of microgrid *I* with the distribution network [*P_i_*(*t*) = (*P*_buy,*i*_(*t*),*P*_sell,*i*_(*t*))]; the shared storage decision variable is the purchased and sold power of shared storage with the distribution network [*P*_SES_(*t*) = (*P*_buy,SES_(*t*),*P*_sell,SES_(*t*))];
(3) Cost: The cost formulas for the distribution network, shared energy storage, and multi-microgrids are Equations (11), (22), and (27), respectively.

The condition for the one-leader-two-followers game to reach equilibrium is as follows:

(40) $$\left\{\begin{array}{l} C_{\mathrm{DN}}[\delta_{\mathrm{DN}}^{*}(t),P_{i}^{*}(t),P_{\mathrm{SES}}^{*}(t)]\leq C_{\mathrm{DN}}[\delta_{\mathrm{DN}}(t),P_{i}^{*}(t),P_{\mathrm{SES}}^{*}(t)]\\ C_{\mathrm{MMG}}[\delta_{\mathrm{DN}}^{*}(t),P_{i}^{*}(t),P_{\mathrm{SES}}^{*}(t)]\leq C_{\mathrm{MMG}}[\delta_{\mathrm{DN}}^{*}(t),P_{i}(t),P_{\mathrm{SES}}^{*}(t)]\\ C_{\mathrm{SES}}[\delta_{\mathrm{DN}}^{*}(t),P_{i}^{*}(t),P_{\mathrm{SES}}^{*}(t)]\leq C_{\mathrm{SES}}[\delta_{\mathrm{DN}}^{*}(t),P_{i}^{*}(t),P_{\mathrm{SES}}(t)] \end{array}\right.$$

Then $\delta_{\mathrm{DN}}^{*}(t),P_{i}^{*}(t),P_{\mathrm{SES}}^{*}(t)$ is considered as an equilibrium solution. A genetic algorithm nested Gurobi solver is used for the solution.

**Figure 3:**
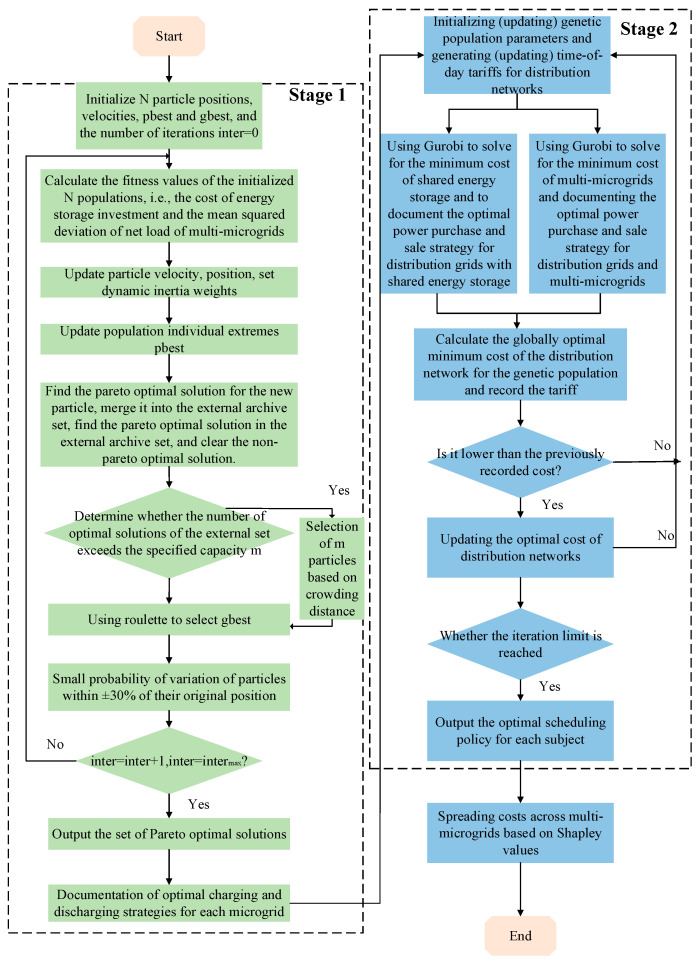
Flowchart for solving the game optimization model.

In Stage 2, a genetic algorithm nested Gurobi solver is used for the solution. The optimization of the time-sharing tariff in the outer distribution network is a nonlinear planning problem, and the genetic algorithm is used for global optimization. The optimization of the interaction decision between shared energy storage and multi-microgrids in the inner layer is linearly transformed into a mixed-integer linear planning problem, which is solved by using the Gurobi solver. In summary, the solution flow of the established one-leader-two-followers game optimization model is shown in Figure 3.

## 4. Calculation of the Capacity of Shared Energy Storage and Power Upper Limits for Charging and Discharging of Shared Energy Storage in a Gaming Framework

There are two methods in the existing studies to calculate the upper capacity limit $E_{\mathrm{SES}}^{\max}$ and upper power limit $P_{\mathrm{SES}}^{\max}$ of shared energy storage: the first is to directly add the upper power limit of shared energy storage demanded by each microgrid as the upper power limit of shared energy storage and directly add the upper capacity limit of shared energy storage demanded by each microgrid as the upper capacity limit of shared energy storage. The second is to take the power limit and capacity limit set by the factory of the energy storage battery as the power limit and capacity limit of the shared energy storage directly.

**Figure 4:**
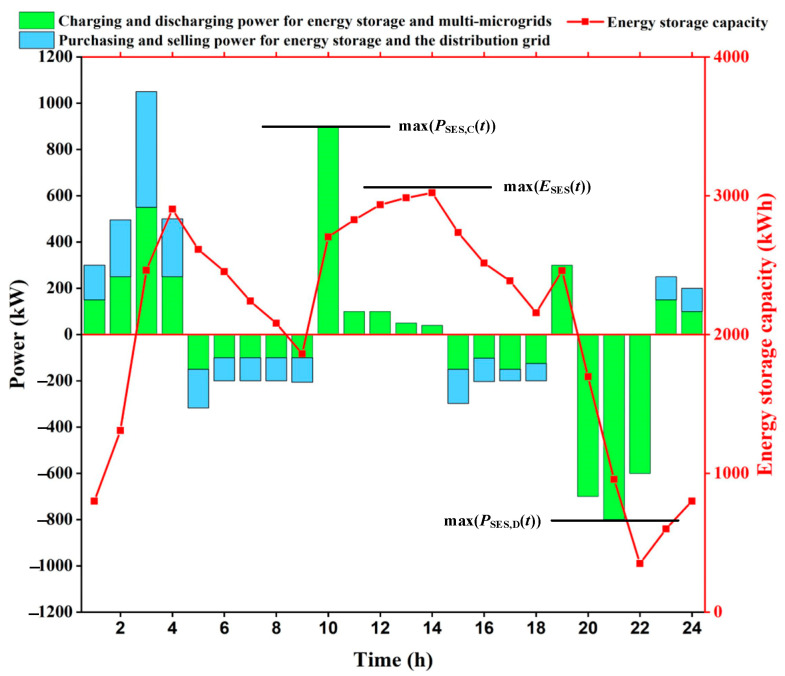
Shared energy storage power and capacity calculation schematic.

In this paper, a new calculation method is proposed, and the calculation principle is shown in Figure 4. Firstly, the charging and discharging power of each microgrid is summed up to calculate the net charging and discharging power, and then the maximum value is filtered to be the upper power limit of the energy storage. Secondly, based on the starting value of the shared energy storage and the net charge/discharge power in each time period, the real-time capacity curve of the shared energy storage is obtained, and then the maximum value of the capacity is obtained, which is then multiplied by the capacity margin to finally arrive at the upper limit of the capacity of the shared energy storage.

As shown in Figure 4, the green bar represents the charging and discharging power of the energy storage with the multi-microgrid, and greater than 0 represents the energy storage charging from the multi-microgrid, and less than 0 represents the energy storage discharging to the multi-microgrid. The blue bar represents the purchase and sale power of the energy storage with the distribution network; greater than 0 represents the energy storage purchasing power from the distribution network, and less than 0 represents the energy storage selling power to the distribution network. The calculation process is as follows:

(41) $$P_{\mathrm{SES}}^{\max}’=\max[P_{\mathrm{SES},C}(t),P_{\mathrm{SES},D}(t)],t=1,\cdots ,T$$

(42) $$E_{\mathrm{SES}}^{\max}’=\mu_{E}\cdot \max(E_{\mathrm{SES}}(t)),t=1,\cdots ,T$$

where $P_{\mathrm{SES}}^{\max}’$ and $E_{\mathrm{SES}}^{\max}’$ are the upper limit of the actual charging and discharging power and the upper limit of the capacity of the energy storage, respectively.

## 5. Case Analysis

### 5.1. Case Parameter Settings

**Figure 5:**
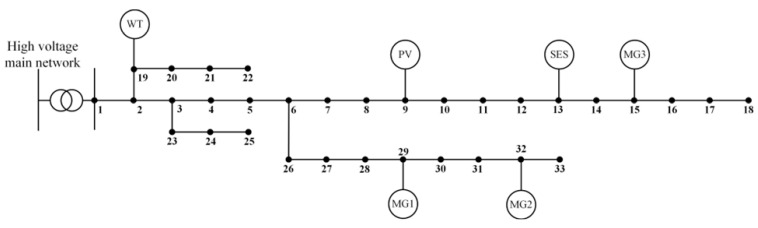
Improved IEEE33 nodal graph for distribution network-multi-microgrids with shared energy storage.

In order to verify the validity of the model and methodology developed in this paper, a simulation test was conducted using the improved IEEE33 node system. As shown in Figure 5, node 1 of the distribution network is connected to the higher-level grid and equipped with a wind farm and a PV plant, which are connected to nodes 19 and 9, respectively. Three microgrids, MG1, MG2, and MG3, are connected to node 29, node 32, and node 15, respectively, and shared energy storage is connected to node 13.

**Figure A1:**
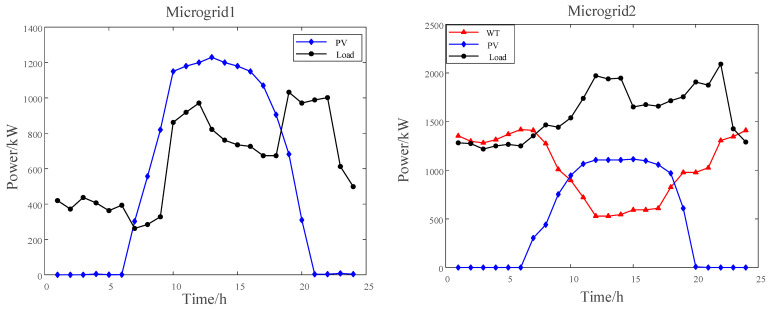

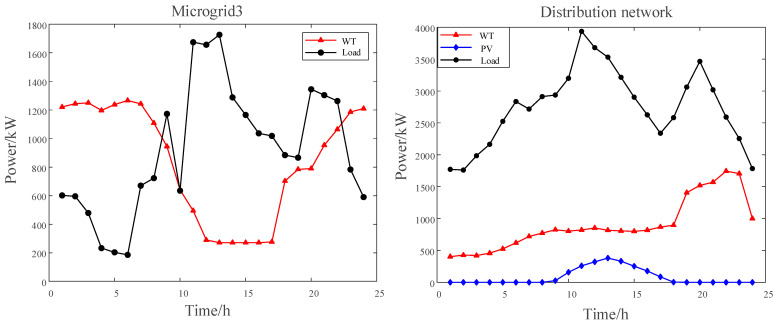
New energy generation and load curves for distribution networks and each microgrid.

The new energy generation and load curves of the distribution network and each microgrid are shown in detail in Appendix A Figure A1, and the specific parameters of the distribution network and shared energy storage are listed in Appendix A Table A1. In the simulation process, the number of populations of the genetic algorithm is set to 20, and the number of iterations is 100.

In the case study of our paper, sensors play a critical role in monitoring and optimizing distribution networks, energy storage systems, and microgrids. The real-time data on power and voltage, essential for our analysis, were accurately captured by sensors embedded in the equipment. These data enable precise real-time decision-making and optimization, enhancing the efficiency and reliability of the integrated energy systems. By leveraging sensor information, we achieve a more responsive and balanced operation of the multi-microgrids, highlighting the transformative impact of sensor technology in modern energy management.

### 5.2. Energy Efficiency Analysis of Game Scheduling for Distribution Networks-Multi-Microgrids with Shared Energy Storage

The proposed method in Section 3.5 is utilized to solve the proposed game model, and the iteration results are shown in Table 2. The results converge when the genetic algorithm is iterated about 90 times. During the iterative process, the distribution network on the subject side, as a leader, usually has more goals and decision-making power, and they are able to strategize and have a significant impact on the overall gaming situation, so their costs are gradually reduced. Shared energy storage and distribution networks, as followers, are subject to the actions and strategies of the leader and can usually only be dispatched according to the leader’s decisions, hence their slightly elevated costs. After the 95th iteration, the costs of all three parties almost no longer change, proving that the strategies of all three parties are the same and they cannot unilaterally adjust their own strategies alone to realize the growth of greater benefits, i.e., the leader’s strategy and the follower’s best-response strategy match each other to reach the equilibrium of the game.

Table 3 shows the time-sharing tariffs set by the distribution network, and the prices in the valley, flat, and peak segments show a gradual upward trend, while the price of electricity sold by the distribution network to the user is always higher than the price of electricity purchased by it in each specific time period, and this pricing mechanism effectively guarantees the economic and stable operation of the distribution network.

In order to verify the validity of the proposed one-master-two-follower game model, the distribution network is taken as the leader, and whether the shared energy storage and multi-microgrids respond to the time-sharing tariffs formulated by the distribution network as the follower is taken into consideration, and three operation schemes are set up for the comparative analysis, as shown in Table 4. Based on the solution results, the tripartite costs of the three operation schemes are compared, as shown in Table 5.

In Scheme 2, the distribution network and the multi-microgrid form a master-slave game. Compared with Scheme 1, the shared energy storage cannot respond to the time-of-day tariff set by the distribution network and cannot utilize its remaining capacity after charging and discharging to the multi-microgrid, so the cost of the distribution network and the shared energy storage is elevated, and the cost of the multi-microgrid is almost unchanged.

In Scheme 3, shared energy storage and multi-microgrids are dispatched separately according to fixed tariffs. Compared with Scheme 1, shared energy storage and multi-microgrids cannot respond to the time-sharing tariffs set by the distribution network, and they can only make adjustments to their own strategy of purchasing and selling electricity to the distribution network, i.e., purchasing electricity when the price of electricity is low and selling electricity when the price of electricity is high so that all three parties’ costs are elevated. Therefore, it is proved that the distribution network as a leader and shared energy storage and multi-microgrids as a follower in Scheme 1, forming a one-master-two-slave game pattern strategy, is most reasonable. The output diagram of each device in each microgrid is shown in Appendix A Figure A3.

### 5.3. Energy Efficiency Analysis of Scheduling with Multiple Microgrids Containing Shared Energy Storage

**Figure A2:**
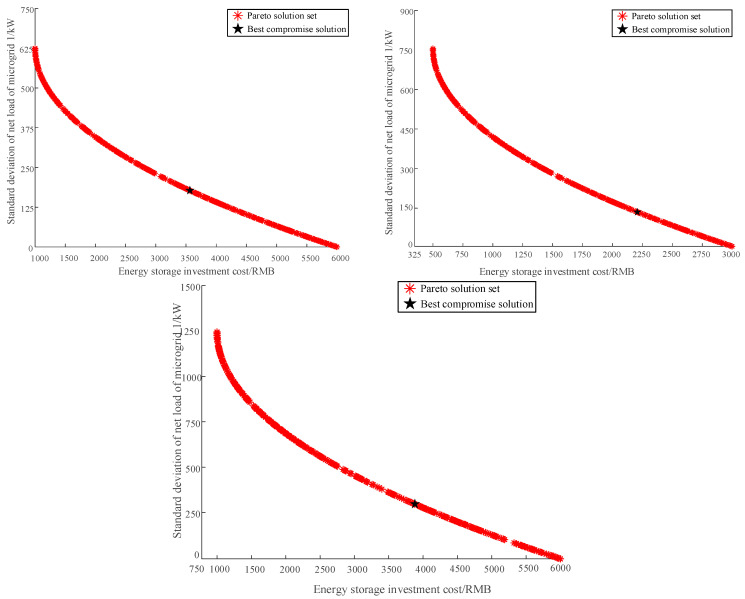
Multi-objective optimization of shared energy storage across microgrids Pareto frontier.

**Figure A3:**
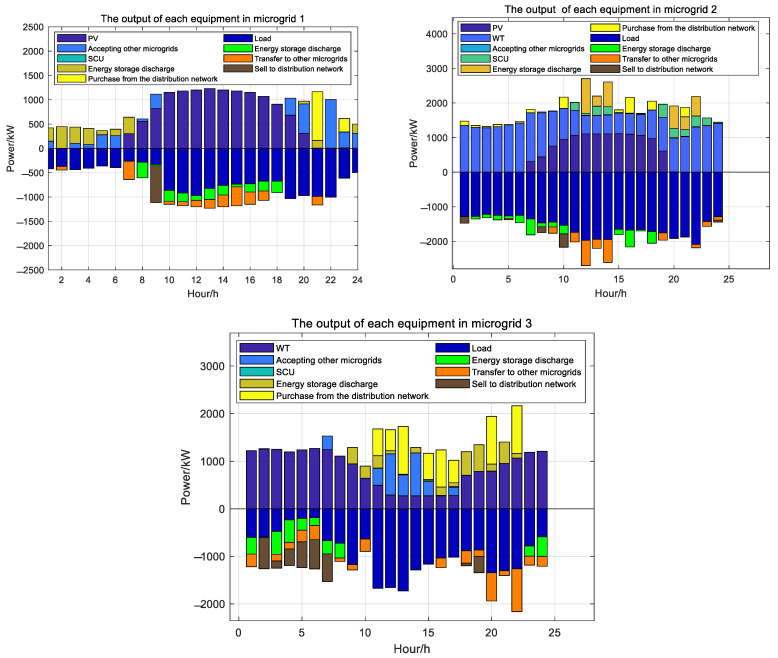
Output diagram of each equipment in each microgrid.

Based on the multi-objective particle swarm algorithm to solve the energy storage allocation model, the final Pareto front is shown in Appendix A Figure A2, from which the optimal compromise solution is selected based on Equations (2) and (3) as the optimal scheduling result.

**Figure 6:**
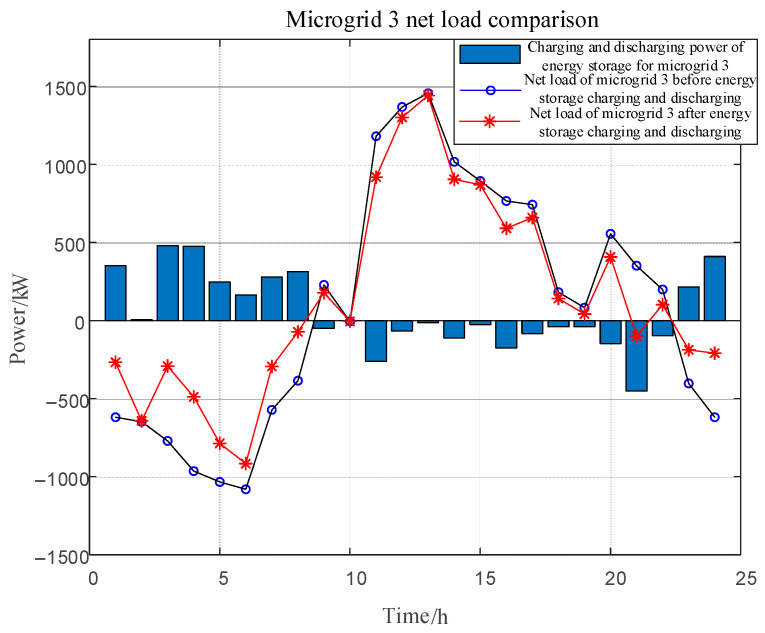
Net load fluctuations before and after sharing energy storage across microgrids.

Figure 6 shows the net load fluctuation before and after sharing energy storage in each microgrid, and Table 6 shows the standard deviation of net load and energy storage investment cost before and after sharing energy storage in microgrids. The blue bar represents the charging and discharging power of the energy storage in microgrid *i*. Greater than 0 means that the energy storage is charging and the net load of the microgrid increases, while less than 0 means that the energy storage is discharging and the net load of the microgrid decreases.

The standard deviation of net load was reduced by 52.9%, 48.6%, and 54.5% for microgrid 1, microgrid 2, and microgrid 3, respectively. It can be seen that the proposed model fully incentivizes the role of energy storage, effectively smoothing out the net load fluctuation caused by the anti-peaking characteristic of the new energy. During the low-load hours, the energy storage system stores power to alleviate the pressure of power abandonment on the grid, while during the peak-load hours, it releases power to fill the power supply gap. This mechanism helps to balance the difference between supply and demand on the grid and significantly improves the stability and reliability of the grid.

**Figure 7:**
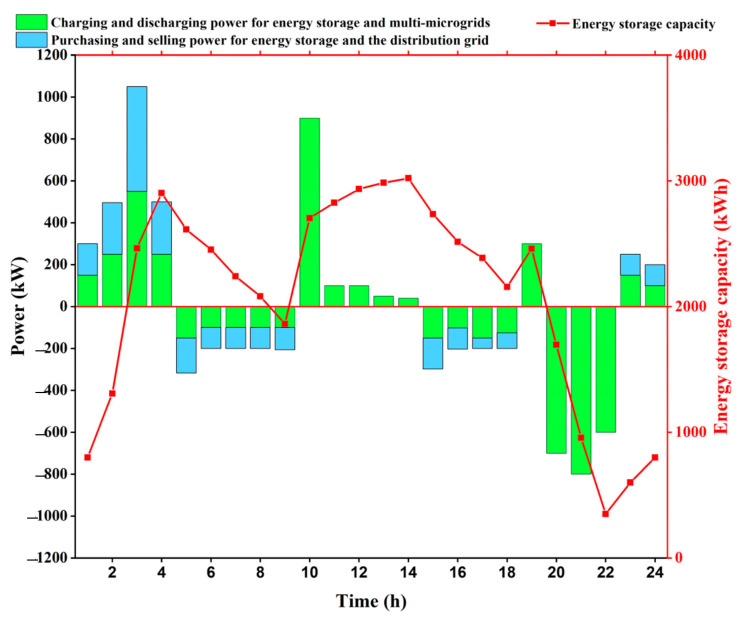
Energy storage charging and discharging power and capacity display.

The energy storage charging and discharging power and capacity are shown in Figure 7. The green bar represents the charging and discharging power of energy storage and multi-microgrid, which is greater than 0 on behalf of energy storage charging from multi-microgrid and less than 0 on behalf of energy storage discharging to multi-microgrid; the blue bar represents the purchasing and selling power of energy storage and distribution network, which is greater than 0 on behalf of energy storage purchasing power from distribution network and less than 0 on behalf of energy storage selling power to distribution network.

The energy storage absorbs power from the multi-microgrids during the valley hours 1:00–4:00 and 23:00–24:00, and since the price of power sold from the distribution network is low at this time, the energy storage purchases power from the distribution network; the energy storage discharges power to the multi-microgrids during the peak hours 5:00–9:00 and 15:00–18:00, and since the price of power purchased from the distribution network is high at this time, the energy storage sells power to the distribution network, 10:00–14:00, 19:00–22:00 are the usual periods, the energy storage does not interact with the distribution network for power, and only meets the charging and discharging demand of the multi-microgrids.

Table 7 shows the comparison of the charging and discharging power and capacity upper limits for the microgrid demand, the actual energy storage, and the energy storage settings. The sum of the upper power and capacity limits of energy storage for each microgrid demand is 1349.32 kW and 5483.09 kW·h, respectively.

Using the new method of calculating the capacity and charging/discharging power upper limit of shared energy storage under the game framework proposed in this paper, the charging/discharging power of each microgrid is aggregated and summed to obtain the net charging/discharging power. When the net charging and discharging power is provided by the shared energy storage, the actual required upper limit of storage power and upper limit of capacity are 846.94 kW and 3021.81 kW·h, respectively, which are smaller than the sum of the upper limit of storage power and upper limit of capacity demanded by each microgrid. Among them, the upper power limit saves 37.23%, and the upper capacity limit saves 44.89%. Meanwhile, it is smaller than the factory-set power and capacity upper limits of the energy storage battery, in which the power upper limit saves 29.42% and the capacity upper limit saves 62.23%. It shows that the provided shared energy storage model can use energy storage resources more efficiently and provides a useful reference for shared energy storage operating companies.

### 5.4. Validity Analysis of Improved Shapley Value Method

The cost comparison between microgrids before and after participating in the alliance is shown in Table 8. The total operating cost after the establishment of the microgrid alliance is RMB 8664.06, which realizes a reduction of 3.6% compared with the total cost of each microgrid operating independently before the alliance. At the same time, the costs shared by each microgrid after the alliance is also reduced compared to the independent operation, which fully proves that the alliance reaches the game equilibrium state and realizes the optimization of economic benefits.

The comparison between the traditional Shapley value method and the improved Shapley value method is shown in Table 9. It can be seen that compared with the traditional Shapley value method, the improved Shapley value method measures the contribution to the coalition through the interaction power between microgrids, and the cost of microgrid 2 is elevated by 14.7% due to the lower interaction power between microgrid 2 and other microgrids, and the cost of microgrids 1 and 3 is reduced by 12.0% and 5.06%, respectively, due to the interaction power between microgrid 1 and microgrid 3 being higher. Power is higher, so the cost of microgrid 1 and microgrid 3 is reduced by 12.0% and 5.06%, respectively, and the overall cost of multi-microgrids is reduced by 1.06%, which not only reflects the advantages of microgrids individually in the coalition but also provides effective ideas for the problem of distributing the benefits of multi-microgrids. At the same time, the solution time was reduced by 134 s, verifying the short-time performance of the improved method.

## 6. Conclusions

In this paper, a cooperative optimization operation model of a distribution network-containing shared energy storage multi-microgrids based on a multi-body game is constructed, aiming to optimize the energy utilization rate of the three parties and improve comprehensive energy efficiency. The model is used for the improved IEEE33 node arithmetic test system, and the following conclusions are drawn by comparing the scheduling results of different operation schemes:

(1) In the first stage, by constructing a multi-objective optimal allocation model and optimization algorithm, the balanced consideration of all objectives is achieved, effectively smoothing out the fluctuation of new energy accessed by each microgrid and reducing the cost of shared energy storage that each microgrid needs to afford.
(2) In the second stage, a one-leader-two-followers game scheduling framework is constructed, which is conducive to promoting cooperation and coordination between the distribution grid, shared energy storage, and multi-microgrids, and realizing the synergy of the three-party scheduling, which can achieve a balanced distribution of the interests of the distribution grid, shared energy storage, and multi-microgrids, and thus increase the overall benefits.
(3) A new method for calculating the upper limit of shared energy storage capacity and charging/discharging power based on a game framework is proposed. Compared with the sum of the energy storage capacity requirements set by each microgrid, the new method is able to realize a saving of 37.23% on the power ceiling and 44.89% on the capacity ceiling; compared with the power and capacity ceilings set by the energy storage batteries at the factory, a saving of 29.42% on the power ceiling and 62.23% on the capacity ceiling is achieved. It shows that the method can be utilized to use energy storage resources more effectively and improve the accuracy of energy storage configuration, thus providing a more economical solution for shared energy storage operating companies.
(4) The Shapley value method is improved by utilizing the interaction power between microgrids to measure their contribution to the coalition. Compared with the traditional Shapley value method, this improvement leads to an increase in the cost of multi-microgrids with lower interaction power and a decrease in the cost of multi-microgrids with higher interaction power, and thus a reduction in the overall cost. The proposed method can fully reflect the individual advantages of microgrids in the coalition, thus providing a strong basis for the distribution of benefits to multi-microgrids.

We plan to focus on several key areas for future research. Firstly, we aim to develop more efficient computational algorithms to address the complexity of the multi-objective optimal allocation model, particularly when dealing with larger and more complex networks. This will enhance the real-time applicability of our model, enabling swift decision-making in practical scenarios. Secondly, we recognize the importance of refining the assumptions and parameters of our game framework to ensure the accuracy of our proposed method for calculating the upper limit of shared energy storage capacity and charging/discharging power. We plan to explore robust validation techniques and adaptive algorithms to improve the reliability of our energy storage configurations. Lastly, we will investigate alternative methods for measuring the interaction power between microgrids, with the goal of refining our improved Shapley value method. By developing more sophisticated interaction metrics, we aim to overcome the challenges associated with accurately quantifying these interactions in complex and diverse energy systems. Overall, these efforts will contribute to advancing the practical application of our energy storage and microgrid optimization strategies.

## Figures and Tables

**Table 1:** Major features of decomposition methods.

Items	Features
Shapley	A fair allocation method in cooperative games, emphasizes individual contributions to the grand coalition, ensuring equitable payoff distribution.
Aumann–Shapley	Extends Shapley to uncertain settings, calculates average contributions across scenarios for fair cost allocation, overcoming elimination order effects.
Maximum–minimum Cost Remaining Saving	A method for minimum-cost maximum flow, optimizing flow to minimize total cost while maximizing flow volume, widely used in network design.

**Table 2:** Genetic algorithm iteration result.

Iteration Number	Distribution Network Cost/RMB	Shared Energy Storage Cost/RMB	Multi-Microgrids Cost/RMB
1	61,212	1963	8523
20	60,954	1986	8546
40	60,533	2013	8561
60	60,128	2032	8586
80	59,865	2076	8626
90	59,624	2125	8647
95	59,624	2147	8664
100	59,624	2173	8664

**Table 3:** Time-of-day tariffs set by the distribution network.

	Time	Tariffs for the Sale of Electricity/(RMB/kWh)	Tariffs for the Purchase of Electricity/(RMB/kWh)
valley	1:00–4:00; 23:00–24:00	0.6249	0.4999
flat	10:00–14:00; 19:00–22:00	0.9561	0.7649
peak	5:00–9:00; 15:00–18:00	1.1000	0.8800

**Table 4:** Comparison of three operation schemes.

Operation Schemes	Is Shared Energy Storage Operating as a Follower?	Are Multi-Microgrids Operating as Followers?
Scheme 1	Yes	Yes
Scheme 2	No	Yes
Scheme 3	No	No

**Table 5:** Comparison of the tripartite costs of the three operating schemes.

Operation Schemes	Costs of the Distribution Network/RMB	Cost of Shared Energy Storage/RMB	Costs of Multi-Microgrids/RMB
Scheme 1	59,624	2173	8664
Scheme 2	60,213	2456	8672
Scheme 3	63,452	3863	10,457

**Table 6:** Comparison of standard deviation of net load and storage investment cost before and after sharing energy storage in microgrids.

Microgrids	Energy Storage Investment Cost/RMB	Standard Deviation of Net Load Before Shared Energy Storage/kW	Standard Deviation of Net Load After Shared Energy Storage/kW
Microgrid 1	3512.63	374.62	176.37
Microgrid 2	2220.65	283.94	146.07
Microgrid 3	3798.02	569.21	259.13

**Table 7:** Comparison of microgrid demand, actual energy storage, and storage-set charging and discharging power and capacity limits.

	Upper Limit of Charge/Discharge Power/(kW)	Upper Limit of Capacity/(kW·h)
Microgrid 1	456.87	2143.04
Microgrid 2	342.14	817.26
Microgrid 3	550.31	2522.79
Energy storage actual	846.94	3021.81
Energy storage set by	1200	8000

**Table 8:** Comparison of costs before and after Microgrid’s participation in the Alliance.

	Before Participation in the Alliance	After Participation in the Alliance
Microgrid 1	2318.09	2149.63
Microgrid 2	3052.88	3021.97
Microgrid 3	3625.92	3492.40
Total	8996.89	8664.06

**Table 9:** Comparison of solution results.

Method	Microgrid	Cost/RMB	Total Cost/RMB	Solution Time/s
Traditional Shapley value method	1	2443.10	8756.99	793
	2	2635.28		
	3	3678.61		
Improved Shapley value method	1	2149.63	8664.00	659
	2	3021.97		
	3	3492.40		

## Data Availability

The data used were shared in our paper.

## Abbreviations

**Abbreviation**	**Description**
A–S	Aumann–Shapley
MCRS	Maximum–minimum Cost Remaining Saving
OM	Operation and maintenance
SES	Shared Energy Storage
WT	Wind Turbine
PV	Photovoltaic
DN	Distribution Network
MG	Microgrid
MMG	Multi-Microgrid
INV	Investigation
SCU	Small coal-fired unit

## Appendix A

**Table A1:** Distribution grid and shared energy storage parameters.

Equipment Parameter Name	Numbers and Units	Equipment Parameter Name	Numbers and Units	Equipment Parameter Name	Numbers and Units
Upper limit of capacity of shared energy storage	8000 kW·h	Charge/discharge O&M cost per unit of power for energy storage	0.1542 RMB/kW	Base voltage	23 kV
Upper limit of power of shared energy storage	1200 kW	Energy storage life	10 Year	Safe voltage range for each node across the network	[0.95,1.05] p.u
Charge/discharge efficiency of energy storage	95%	System baseline capacity	100 MVA	Upper limit of branch circuit transmission power capacity	3000 kW
Investment cost per unit capacity of energy storage	1250 RMB/(kW·h)	Investment cost per unit of power for energy storage	4375 RMB/kW	Capacity margins for shared energy storage in microgrids	1.25
